# Suicidal transition rates and their predictors in the adult general population: a repeated survey over 21 years in France

**DOI:** 10.1192/j.eurpsy.2024.1782

**Published:** 2024-10-29

**Authors:** Fabrice Jollant, Christophe Leon

**Affiliations:** 1 Faculté de médecine, Université Paris-Saclay, Le Kremlin-Bicêtre, France; 2 Department of Psychiatry, Bicêtre Hospital, AP-HP, Le Kremlin-Bicêtre, France; 3 Department of Psychiatry, CHU Nîmes, Univ Montpellier, Nîmes, France; 4 Department of Psychiatry and McGill Group for Suicide Studies, McGill University, Montréal, QC, Canada; 5 Health Promotion and Prevention Division, Mental Health Unit, Santé Publique France, Saint-Maurice, France

**Keywords:** attempt, general population, suicide, survey, transition, youth

## Abstract

**Background:**

The “suicidal transition” from ideation to an act has become a specific topic of research. However, rates in the general population, variations across time and risk factors are unclear.

**Methods:**

Data were collected from the phone survey *Baromètre Santé* among 18–75-year-olds in France. Seven independent samples interviewed between 2000 and 2021 (total N = 133,827 people; 51.3% females) were questioned about suicidal ideation and attempts over the previous 12 months. Transition was calculated as the weighted ratio of attempt on ideation 12-month rates.

**Results:**

Mean 12-month rates of suicidal ideation, attempts and transition were 4.7% (95% Confidence Interval (CI) (4.6–4.8)), 0.5% (95% CI (0.4–0.5)) and 7.7% (95% CI (6.8–8.6)), respectively. Transition rates varied between 4.5 and 11.9% across surveys. In multivariable analyses, higher transitions rates were associated with a previous suicide attempt (adjusted Odds Ratio (aOR) = 11.1 95% CI (7.9–15.6)); 18–25 vs 26–55-year-olds (1.8 95% CI (1.2–2.8)); lower vs higher income (1.7 95% CI (1.0–2.7); and lower vs higher professional categories (aOR around 1.9). No significant association was found with gender, education level, employment status, living alone, urbanicity, current major depression, daily smoking, weekly heavy drinking, cannabis use, and body mass index.

**Conclusions:**

Most people with suicidal ideation do not attempt suicide. These findings emphasize the need to avoid generic terms such as “suicidality”, and to increase research on suicidal transition to improve prevention and prediction. They may also inform the organization of suicide prevention in the general population.

## Introduction

Over recent years, the suicidal transition, that is the process leading a person with suicidal ideation to carry out a suicidal act, has gained growing attention in suicidology. In contrast to the concept of “suicidality”, where suicidal ideation and suicidal acts are considered together, the suicidal transition suggests a clear distinction between ideas and acts in terms of phenotypes, risk factors and mechanisms.

A first argument in favor of the suicidal transition concept is given by descriptive epidemiology: Most people with suicidal ideation do not attempt or die from suicide. For instance, the 12-month prevalence in France in 2021 among adults was 4.2% for suicidal ideation and 0.5% for suicide attempts [1], while 0.013% of the population died from suicide [2]. In the National Comorbidity Survey (NCS) in the USA, 12-month prevalence for suicidal ideation and attempts in adults was 2.6 and 0.4%, respectively [3]. In the World Health Surveys conducted by the World Health Organization, corresponding prevalences were 2.0 and 0.3% in high-income countries, and 2.1 and 0.4% in low- and middle-income countries [4]. A recent review of the literature of 18 longitudinal studies in non-clinical populations has estimated the suicidal transition rate, that is the rate of people who attempt suicide among those with suicidal ideation, between 2.6 and 37% [5].

Another argument supporting suicide transition is the poor value of suicidal ideation to predict suicide. While suicidal ideation and acts are significantly associated at the statistical level, a meta-analysis of 71 studies [6] reported a positive predictive value between 0.3 and 3.9% and a sensitivity between 22 and 46%. Thus, suicidal ideation alone appears to have limited utility to predict suicide. Yet, the identification of suicidal ideation critically impacts medical decisions, including hospitalization and involuntary admission. Identifying factors more specifically associated with the suicidal transition in those with suicidal ideation may improve clinical prediction and practice.

A third argument is a distinction between suicidal ideation and acts in terms of related risk factors and mechanisms. So far, these arguments remain weak. Based on the NCS, Nock et al. [7] showed that while depression was associated with suicidal ideation, it was not associated with suicide attempt in those with suicidal ideation. In contrast, disorders characterized by high anxiety and agitation or poor impulse control were significantly associated with acting out. However, these findings were not supported by all studies [5].

Beyond mental disorders, other factors influence the emergence of suicidal ideation or the suicidal transition. Preliminary findings suggest that some neurocognitive processes differ between suicide attempters and suicidal ideators. Wagner et al. [8] reported significant differences in brain activation at rest between the two populations. Interestingly, there were almost no differences between ideators and non-suicidal depressed patients in this study. Moreover, a meta-analysis of neurocognitive tests [9] found that poor cognitive inhibition and risky decision-making distinguished attempters from ideators, but not ideators from non-ideators. These cognitive functions may, therefore, contribute more specifically to the suicidal transition during a suicidal crisis. More studies are needed to confirm these preliminary findings.

In summary, the concept of suicidal transition is appealing but deserves more research. In this study, our objective was to investigate the suicidal transition at the epidemiological level based on data from a large and recurring national survey among adults, the *Baromètre Santé* run by Public Health France. As Haregu et al. showed [5], few studies have been conducted in general populations, and none were repeated to investigate shifts in transition rates across time.

More specifically, we wished to 1) estimate the suicidal transition rate in a French adult general population; 2) investigate potential variations in suicidal transition rates across seven independent surveys over 21 years including pre- and post-covid-19 pandemic; and 3) identify potential sociodemographic and clinical risk factors of suicidal transition in people with suicidal ideation.

## Methods

### Data source and population

This study was based on cross-sectional data from the *Baromètre Santé* survey run by Santé Publique France (Public Health France). The *Baromètre Santé* survey is regularly conducted in independent samples representative of the French adult general population. The target population in this study includes French-speaking people aged 18 to 75-year-old living in mainland France.

For the 2021 survey (the most recent survey), the method was based on a random generation of landline and mobile telephone numbers. Participants were selected using a two-stage landline survey (selection of one individual per household using the Kish method [10] and a one-stage mobile survey (selection of the person answering the phone). It was conducted by telephone by *Institut Ipsos* from February 11 to December 15, 2021 (with a summer break from July 19 to August 22). A total of 24,514 people were interviewed, 17,496 mobile phones (71%) and 7,018 on landlines (29%). The participation rate was 44.3% for a questionnaire lasting an average of 36 minutes [11].

Data from previous surveys in 2000, 2005, 2010, 2014, 2017 and 2020 were also analyzed to assess potential changes in transition rates over time. These surveys used the same methodology as the *Baromètre Santé 2021.* Importantly, participants are not the same across surveys.

The French National Commission for Information and Freedom (CNIL) approved the survey protocol. All participants gave informed consent.

### Main variables

Suicidal ideation were investigated with the following question: “*In the last twelve months, have you thought about suicide?*” (possible answers: Yes or No). Participants can opt to not answer this question. Suicide attempt history was first investigated with the following question: “*Have you ever attempted suicide?*” (Yes/No). If yes: “*How many times has this happened to you?*” (minimum 1- maximum 50). “*If it’s not too difficult for you, we’re going to talk about the last time you attempted suicide. Did this attempt take place in the last twelve months?*” (Yes/No/no answer).

### Additional variables

We collected the following additional variables to investigate risk factors of the suicidal transition: gender (male / female), age (in 3 categories: 18–25 / 26–55 / 56–75 year-old), education level (secondary school or lower / university level or equivalent), self-reported incomes (divided in three terciles: lower / median / higher), employment status (working or studying / unemployed / inactive or retired), professional activity (farmers, craft workers, shopkeepers and small business owners / managers and higher intellectual professions / intermediate professions / employees / manual workers / others, refusal or no answer), living alone (yes / no), urbanicity (urban / rural), a previous suicide attempt (that is a suicide attempt in addition to the one that occurred during the last 12 months; this act may have or not taken place during the last 12 months; of note, if suicide attempts only occurred before the last 12 months, this was not considered a previous suicidal act) (yes / no), current major depressive episode (yes / no) based on the short version of the CIDI-SF questionnaire [12], daily smoking (yes / no), weekly heavy episodic drinking defined as at least 6 standard (10 grams) alcoholic drinks on a single occasion at least once a week during the last 12 months (yes / no), use of cannabis at least once over the last month among 18–65-year-olds (yes / no), and current Body Mass Index (Underweight (≤18), normal weight (19–24), overweight (25–29), obesity (≥30)).

### Statistical analyses

The *Baromètre Santé* relied on sampling weights, followed by post-stratification to correct the sample on non-response and multiple adjustment criteria from auxiliary information, which was obtained from the most recent external national census data information. Sampling weights were calculated from an individual’s probability of inclusion from three sources of information: 1) the probability of selecting the telephone number, 2) the number of landlines and mobile phone numbers, and 3) the number of individuals from whom the selection was made. The probability of selecting an individual, while ignoring the probability of being surveyed multiple times, was obtained by summing for each respondent the telephone numbers, the probability of selecting the telephone number divided by the number of eligible individuals reachable on that telephone number. In addition, each survey year accounted for non-response through post-stratification. Post-stratification involved modifying the weight of each respondent to correct potential sampling errors using auxiliary information available in the Employment Survey conducted by the National Institute of Statistics and Economic Studies (INSEE), such as: gender crossed with age in decade intervals, size of urban unit, region of residence, level of education, and number of persons in the household. The adjusted sample thus matched the structure of the overall population, ensuring the representativeness of the sample.

Data were analyzed both by year and pooled from the seven surveys. Descriptive analyses were performed to characterize the sample and to present the prevalence of 12-month rates of suicidal thoughts, suicide attempts and suicidal transition by year of survey and overall, as the mean over seven years. The suicidal transition rate was calculated as the rate of people who reported a suicide attempt over the last 12 months among those who reported suicidal ideation over the last 12 months.

For qualitative variables, group comparisons were performed with Chi square tests and univariate logistic regressions. A multivariable logistic regression was then conducted using variables significantly associated with 12-month suicidal transitions in univariate analysis at 5%. These associations were assessed by adjusted odds ratios (aOR) and presented with their 95% confidence intervals (CI).

All analyses were performed using Stata® (version 15.0 SE).

## Results

### Overall rates and changes over 21 years

**Figure 1.**
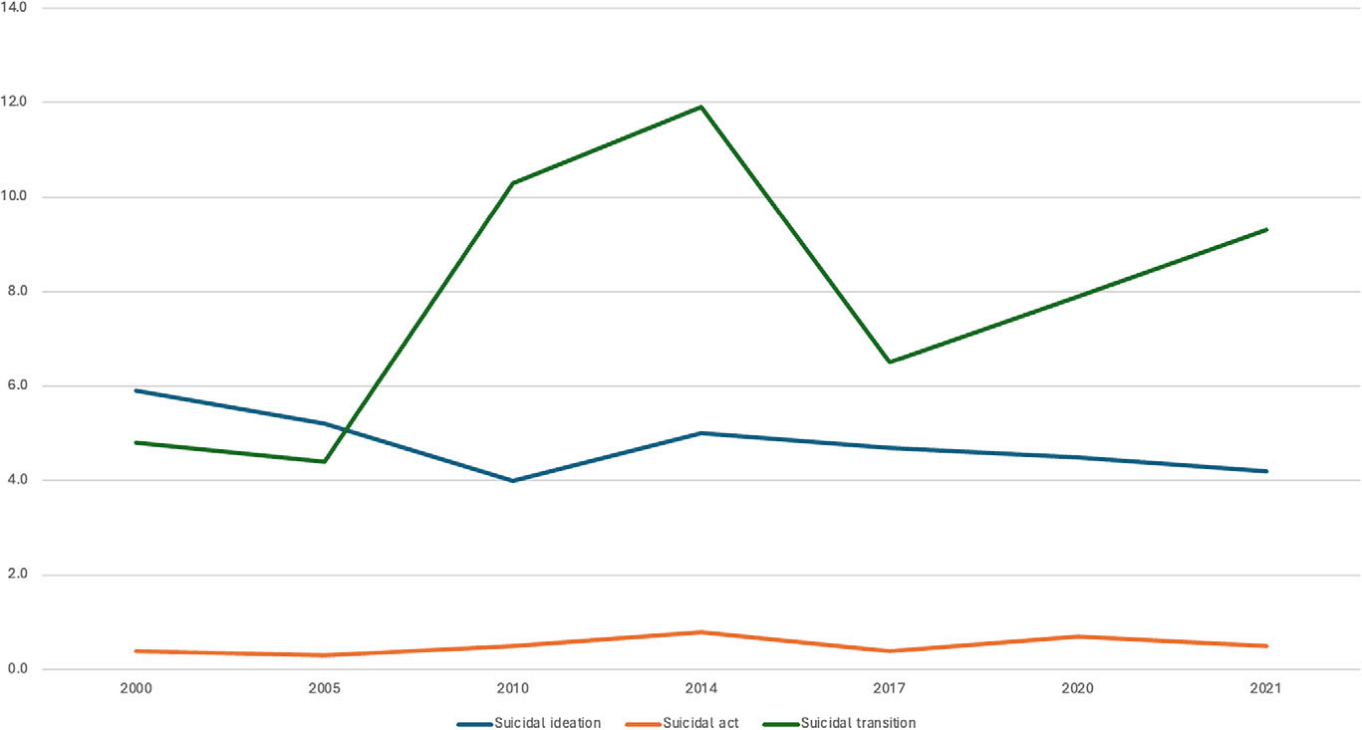
Variations in twelve-month rates (in %) of suicidal ideation, suicide attempts, and suicidal transition over the seven surveys between 2000 and 2021.

Overall, 133,827 people (females: 51.3%) were interviewed across seven surveys between 2000 and 2021. Data were available for suicide ideas in 133,661 individuals, for suicide attempt in 133,519 and for both in 133,416 (99.7% of the whole population). For each of the seven surveys, the number of people who answered the questions, 12-month rates of suicidal ideation and suicide attempts, and suicidal transition are reported in Table 1 and Figure 1.

**Table 1.** Twelve-month rates of suicidal ideas, suicide attempts and suicidal transition over the seven surveys between 2000 and 2021, and in total

	Suicidal ideas		Suicide attempt		Transition rate
Year	Total respondants	*N* (%)	Total respondants	*N* (%)	% (95% CI)
2000	12,578	844 (5.9)	12,580	74 (.4)	4.8 (3.4–6.8)
2005	24,571	1,399 (5.2)	24,570	53 (.3)	4.4 (3.2–6.1)
2010	25,021	1,088 (4.0)	25,000	127 (.5)	10.3 (8.4–12.7)
2014	15,169	734 (5.0)	15,147	89 (.8)	11.9 (9.0–15.6)
2017	25,295	1,148 (4.7)	25,251	75 (.4)	6.5 (4.8–8.7)
2020	8,451	357 (4.5)	8,436	29 (.7)	7.9 (4.4–13.9)
2021	22,576	886 (4.2)	22,535	90 (.5)	9.3 (7.1–12.1)
Total	133,661	6,456 (4.7)	133,519	537 (.5)	7.7 (6.8–8.6)

Abbreviation: 95% CI, 95% confidence interval.

*Note*: All percentages are weighted.

We examined the total group of 166 people (0.1%) who did not answer about 12-month suicidal ideation and compared them to the rest of the sample. There was no significant difference in terms of age, gender, or urbanicity. However, they less often reached higher education (university or equivalent: 15.5 vs 29.6%), had lower incomes (lowest tercile: 42.4 v 32.4%), were less often working (38.7 vs 55.8%) and were more often living alone (36.5 vs 15.9%).

The mean 12-month rate of suicidal ideation over 21 years was 4.7% (95% CI (4.6–4.8), ranging from a minimum of 4.0% in 2010 and a maximum of 5.9% in 2000. There were significant changes over time (Chi2 = 92.8; p < .0001). Comparison of Year n and Year n-1 showed a significant decrease in 2005 and 2010 followed by a significant increase in 2014.

The mean 12-month rate of suicide attempts was 0.5% (95% CI (0.4–0.5) ranging from 0.3% in 2005 to 0.8% in 2014. There were significant changes over time (Chi2 = 65.0; p < .0001) with significant increases in 2010 and 2014 followed by a significant decrease in 2017 and a re-increase in 2020.

The mean suicidal transition rate over 21 years was 7.7% (95% CI (6.8–8.6). It varied between 4.5% in 2005 and 11.9% in 2014 with significant changes over time (Chi2 = 63.5; p < .0001) including a significant increase in 2010 and a significant decrease in 2017. Of note, we observed a non-significant increase between 2017 and 2020 / 2021 (Chi2 = 5.4; p = 0.08), that is pre vs post Covid-19 pandemic.

### Factors associated with variations in transition rates

**Figure 2.**
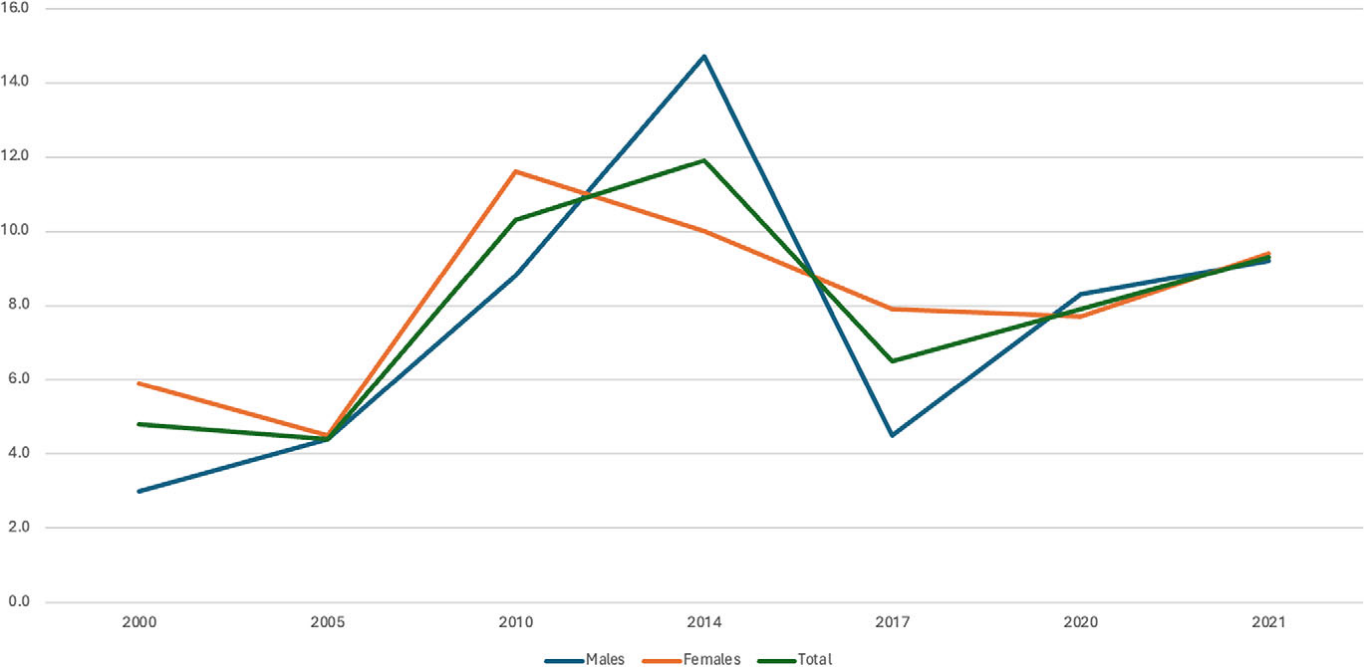
Variations in twelve-month suicidal transition rates (in %) in males and females and in total over the seven surveys between 2000 and 2021.

Regarding gender, mean suicidal transition rates were 7.2% (95% CI (5.9–8.6)) in males (min = 3.0; max = 14.7) and 8.0% (95% CI (6.9–9.2)) in females (min = 4.5; max = 11.6) without significant gender difference in any year or overall (Table 2 and Figure 2). Significant changes over time were observed in males (Chi2 = 46.8; p = 0.0001), with significant increases in 2010 and 2014 followed by a significant decrease in 2017; and in females (Chi2 = 30.1; p = 0.02) with a significant increase in 2010 followed by a slower and non-significant decrease. Again, non-significant increases were observed between 2017 and 2020, notably in males.

**Table 2.** Twelve-month suicidal transition rates in males and females over the seven surveys between 2000 and 2021 and in total, and group comparisons

	Males		Females		Group comparison
Year	*N*	% (95% CI)	*N*	% (95% CI)	Chi-2 (*p*)
2000	297	3.0 (1.6–5.4)	546	5.9 (3.9–8.8)	3.6 (0.07)
2005	520	4.4(2.5–7.7)	876	4.5 (3.1–6.4)	0.0 (0.9)
2010	412	8.8 (6.1–12.5)	668	11.6 (8.8–14.9)	2.2 (0.2)
2014	295	14.7 (9.9–21.4)	431	9.9 (6.6–14.4)	4.0 (0.2)
2017	426	4.5 (2.6–7.9)	712	7.9 (5.6–11.1)	5.2 (0.1)
2020	152	8.3 (3.6–17.7)	202	7.7 (3.3–16.9)	0.0 (0.9)
2021	355	9.2 (6.1–13.6)	521	9.4 (6.6–13.3)	0.0 (0.9)
Total	2,457	7.2 (5.9–8.6)	3,956	8.0 (6.9–9.2)	1.6 (0.3)

Abbreviation: 95% CI, 95% confidence interval.

*Note*: All percentages are weighted. *N* are the number of individuals with suicidal thoughts.

**Figure 3.**
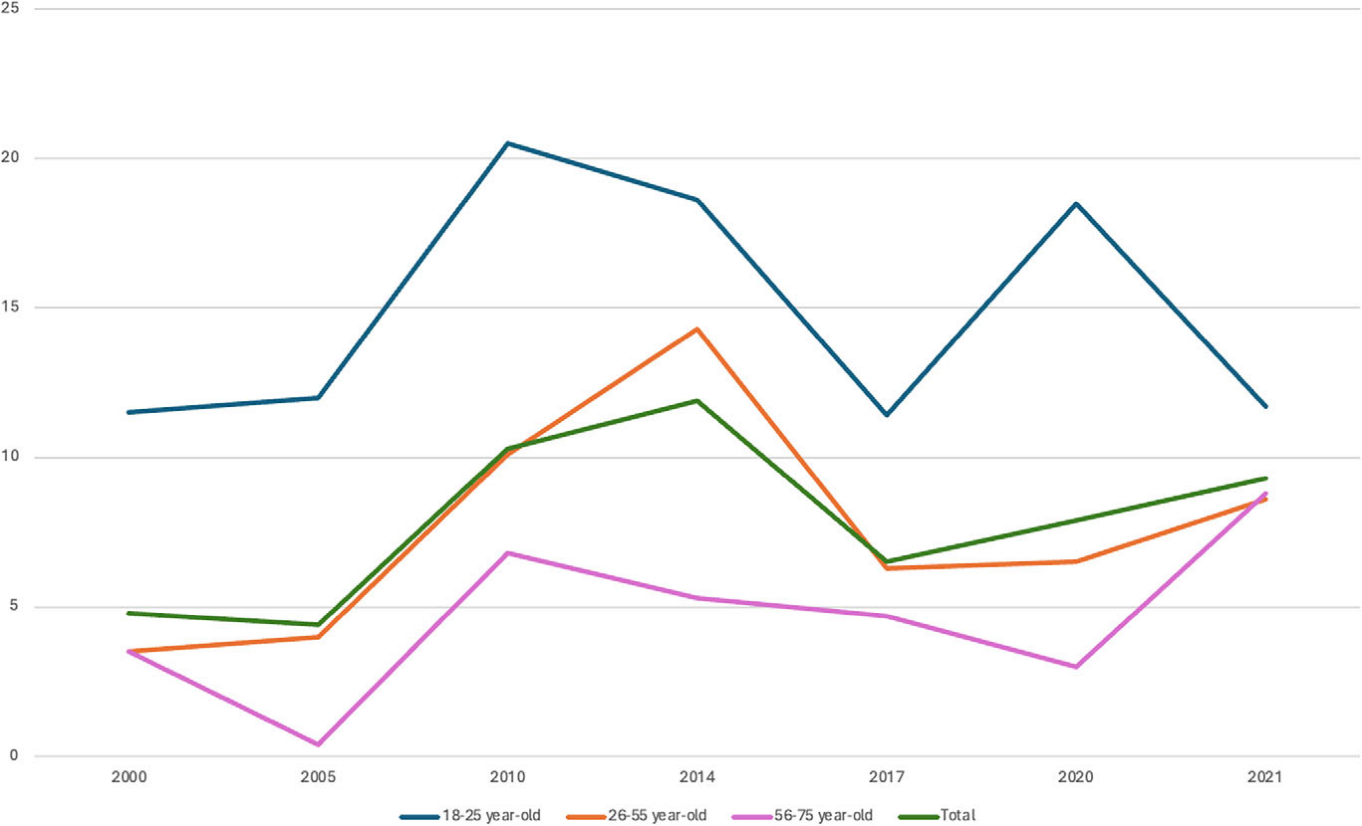
Variations in twelve-month suicidal transition rates (in %) in the three age groups and in total over the seven surveys between 2000 and 2021.

Transition rates varied across age groups. They were higher in 18–25-year-olds in five of the seven surveys and in total (Table 3 and Figure 3). Mean transition rates were 13.8% in 18–25-year-olds, 7.3% in 26–55-year-olds, and 4.8% in 56–75-year-olds. There was no significant change over time in 18–25-year-olds (Chi2 = 7.7; p = 0.5); significant changes over time in 26–55-year-olds (Chi2 = 66.4; p < .00001), with a significant increase in 2010 and decrease in 2017; and significant changes over time in 56–75-year-olds (Chi2 = 27.6; p = 0.01), with a significant decrease in 2005 and increase in 2010.

**Table 3.** Twelve-month suicidal transition rates (in %) in the three age groups over the seven surveys between 2000 and 2021 and in total, and group comparisons

	18–25-year-old		26–55-year-old		56–75-year-old		Comparison
Year	*N*	% (95% CI)	*N*	% (95% CI)	*N*	% (95% CI)	Chi-2 (*p*)
2000	127	11.5 (6.7–19.1)	555	3.5 (2.1–5.7)	161	3.5 (1.1–10.4)	16.6 (0.006)
2005	157	12.0 (7.2–19.3)	920	4.0 (2.6–6.0)	319	0.4 (0.1–1.6)	41.2 (<.0001)
2010	95	20.5 (12.9–30.9)	650	10.1 (7.7–13.1)	335	6.8 (3.9–11.7)	17.0 (0.005)
2014	60	18.6 (10.1–31.7)	419	14.3 (10.0–19.9)	247	5.3 (2.9–9.4)	14.5 (0.005)
2017	120	11.4 (5.8–21.0)	653	6.3 (4.4–9.0)	365	4.7 (2.2–9.8)	7.1 (0.2)
2020	53	18.5 (8.0–37.2)	174	6.5 (2.3–16.8)	127	3.0 (1.0–8.2)	15.6 (0.03)
2021	144	11.7 (7.1–18.7)	482	8.6 (5.8–12.5)	250	8.8 (5.0–15.2)	1.6 (0.6)
Total	756	13.8 (11.1–17.0)	3,853	7.3 (6.3–8.5)	1804	4.8 (3.6–6.4)	70.3 (<.0001)

Abbreviation: 95% CI, 95% confidence interval.

*Note*: All percentages are weighted. *N* are the number of individuals with suicidal thoughts.

There was no significant age x gender interaction overall (p = 0.07).

Finally, we investigated factors associated with the suicidal transition by pooling data from the most recent surveys (2010, 2014, 2017, 2020 and 2021). Findings are reported in Table 4. In multivariable analyses, including gender and variables statistically significant at 5% in univariate analyses, significant associations were found for past suicide attempts, year of survey, age group, income and category of professional activity.

**Table 4.** Sociodemographic and clinical factors associated with the suicidal transition rate in univariate and multivariable analyses (pooled data from 2010 to 2021)

Variables	Suicidal transition rate %	Univariate analyses		Multivariable analyses
	(95% CI)	Chi^2^	*p*	Adjusted OR (95% CI)
Overall (2010–2021)	9.1 (8.0–10.3)	–	–	
*Year of survey* ^*^		19.0	0.05	
2010	10.4 (8.4–12.8)			**1.3 (1.0–1.7)** ^a^
2014	11.9 (9.0–15.6)			**1.4 (1.0–2.0)** ^a^
2017	6.5 (4.8–8.7)			**0.7 (0.5–1.0)** ^a^
2020	7.9 (4.4–13.9)			0.9 (0.6–1.5)
2021	9.3 (7.1–12.1)			1.0 (0.6–1.2)
*Gender*		0.6	0.5	
Male	8.7 (7.1–10.6)			– 1 –
Female	9.4 (8.0–11.6)			0.9 (0.6–1.2)
*Age*		37.4	<0.0001	
18–25	14.9 (11.4–19.1)			**1.8 (1.2–2.8)** ^b^
26–55	9.2 (7.7–10.8)			– 1 –
56–75	6.0 (4.4–8.0)			0.8 (0.5–1.2)
*Education level*		9.3	0.01	
Secondary school or lower	9.9 (8.6–11.5)			– 1 –
University level or equivalent	6.8 (5.3–8.7)			1.3 (0.9–1.9)
*Income*		42.4	<0.0001	
Lower	11.5 (9.7–13.6)			**1.7 (1.0–2.7)** ^a^
Median	7.4 (5.7–9.4)			1.4 (0.8–2.2)
Higher	4.4 (3.1–6.1)			– 1 –
Undisclosed	12.6 (7.9–19.5)			1.9 (0.9–3.9)
*Employment status*		17.2	0.01	
Working	7.3 (6.0–8.9)			– 1 –
Unemployed	12.4 (9.1–16.6)			1.2 (0.7–2.0)
Inactive	9.9 (8.1–12.0)			1.0 (0.7–1.5)
*Professional activity*		31.0	0.005	
Farmers, craft workers, shopkeepers and small business owners	9.0 (5.6–14.1)			2.0 (0.9–4.2)
Managers and higher intellectual professions	4.3 (2.8–6.5)			– 1 –
Intermediate professions	7.9 (5.8–10.8)			**1.9 (1.0–3.4)** ^a^
Employees	10.2 (8.3–12.6)			**1.8 (1.0–3.1)** ^a^
Manual workers	10.1 (7.9–12.8)			**1.9 (1.1–3.5)** ^a^
Others, refusal, no answer	18.6 (9.3–33.9)			2.3 (0.9–6.3)
*Living alone*		1.6	0.3	
No	8.7 (7.4–10.3)			
Yes	9.9 (8.1–12.1)			
*Urbanicity*		2.3	0.2	
Rural	7.8 (6.0–10.2)			
Urban	9.5 (8.2–10.9)			
*Previous suicide attempt*		20.8	<0.0001	
No	3.8			– 1 –
Yes	30.9			**11.1 (7.9–15.6)** ^c^
*Current major depressive episode* ^**^		0.4	0.6	
No	7.5			
Yes	8.3			
*Daily smoking*		37.7	<0.0001	
No	6.8 (5.6–8.2)			– 1 –
Yes	12.3 (10.4–14.6)			1.4 (0.98–1.9)
*Weekly heavy drinking*		11.8	0.01	
No	8.6 (7.5–9.8)			– 1 –
Yes	13.7 (9.6–13.1)			1.1 (0.7–1.8)
*Monthly Cannabis use*		1.9	0.3	
No	9.4			
Yes	11.3			
*Body Mass Index*		3.8	0.6	
Underweight	12.0 (7.3–19.3)			
Normal	8.7 (7.3–10.4)			
Overweight	8.7 (6.7–11.2)			
Obesity	10.0 (7.1–13.8)			

Abbreviations: OR, odd ratio; 95% CI, 95% confidence interval.

a p < 0.05

b p < 0.01

c p < 0.001

* Adjusted OR calculated vs mean of the 2010–2021 period.

** Data limited to 2010, 2017 and 2021.

## Discussion

Our study investigated the suicidal transition rate in 133,661 French adults living in mainland France, of whom 6,456 reported past 12-month suicidal ideation. It represents one of the largest studies published on the topic. We found mean suicidal transition rates of 7.7% (95% CI (6.8–8.6)) in the French adult general population across seven independent samples interviewed between 2000 and 2021. These rates varied over 21 years between a minimum of 4.5% in 2005 and a maximum of 11.9% in 2014. Of note, a non-significant increase in suicidal transition rates was found after the Covid-19 pandemic started in early 2020, from 6.5% in 2017 to 7.9% in 2020 and 9.3% in 2021. Several factors were significantly associated with the transition rate. The factor associated with the highest risk of suicidal transition was a previous suicide attempt (aOR = 11.1). Younger age (18–25-year-olds) showed the highest transition rates across age groups with a mean transition rate of 13.8%, while older ages (56–75-year-olds) showed the lowest (mean = 4.8%). In contrast, there was no gender difference. Additional independent sociodemographic factors were lower income and lower and intermediate professional categories.

The study had several limitations. First, main outcomes (suicide ideation and attempt) were based on self-reports in a phone interview. Memory and desirability bias may have affected reports of these variables. Moreover, no definition of suicidal ideation and suicide attempt was given during the interview, which may have led to heterogeneity in measures. Second, the design of the study was retrospective. A longitudinal study may best capture the temporal sequence from suicidal ideation to an act although our findings appear to be within range of previous longitudinal cohort studies as discussed below. Third, while the number of participants was high, the number of people who attempted suicide was relatively limited (overall N = 537), which may have reduced the necessary statistical power to identify particular risk factors. Fourth, the *Baromètre Santé* does not include adolescents, a population with a high rate of self-harm [13] as well as people currently hospitalized in psychiatry and inmates. Fifth, many relevant variables are not available including family history, exhaustive mental disorder history, recent life events, and treatments. Sixth, 0.1% of the interviewed people refused to answer about 12-months suicidal ideation. They appeared to be less socially favored than those who answered in terms of incomes, education, work and marital status. Among these four variables, only lower income was associated with higher transition rates. Therefore, our findings related to transition rates may be slightly underestimated.

The mean suicidal transition rate found here is within the range (2.6–37%) of a recent review of 18 longitudinal studies in non-clinical populations [5]. In a comparable study in the USA (adult general population with 12-month follow-up using mailed questionnaires), the transition rate was higher, reaching 15.7% (10.6–20.8) [3]. In a Dutch adult population followed for 36 months, the transition rate was 12.2% (11.2–13.2) [14]. In a US adult cohort followed for 156 months, the transition rate was 10.3% (3.6–17) [15]. Much higher rates (above 20%) were rare and found in specific populations, including adolescents and army veterans [5]. Overall, our study adds to an unequivocal literature showing that a large majority (above 80% and usually 90%) of people with suicidal ideation in the general population do not attempt suicide.

In our study, we compared transition rates across different years in independent samples. Mean transition rates varied across surveys between a minimum of 4.5% in 2005 and a maximum of 11.9% in 2014. However, an explanation may be that small changes in 12-month self-reported rates of suicidal ideation and suicide attempt may have a large impact on the ideation/attempt ratio. For instance, a significant increase in transition rates was observed in 2010 and a significant decrease in 2017. The 2010 increase (vs 2005) was related to a combination of an increase of 0.2% in suicide attempt rate and a decrease of 1.2% in suicidal ideation rate while the 2017 decrease (vs 2014) was related to a decrease of 0.4% in suicide attempt rate along a limited decrease of 0.3% in suicidal ideation rate. In these conditions, it is difficult to conclude that some periods in time (here, somewhere between 2005 and 2017) may have had an impact of the suicidal transition process *per se.* While variations in suicidal rates seem to be related to variations in self-reported measures of suicidal ideation and attempts, no clear changes in suicidal transition rates appeared over the last 21 years and no clear temporal trends. Therefore, following the temporal changes in suicidal transition rates may usually not be relevant.

The strongest risk factor of suicidal transition was by far a previous suicide attempt with an increased risk multiplied by 11 as compared to people with no such history. This is in line with an important literature [16, 17]. Two non-exhaustive mechanisms may be in play. A past attempt may reveal the existence of long-term vulnerability factors enabling acting out in conditions of suicidal ideation and stress. The experience of a past suicide attempt may, in some individuals, also facilitate the realization of another act when suicidal following a process described as “acquired capability” or increased facilitation [18].

While gender does not seem to affect transition rates as previously reported [3, 14, 15], age contributed to significant differences. In our study, young adults below 25 were notably associated with higher rates over the seven surveys and overall, with an aOR of 1.8 as compared to middle-aged adults. However, these findings are conflicting. Cohorts with adolescents have shown low [19, 20], to very high [21, 22] suicidal transition rates. Borges et al. [3] found a trend toward higher rates in younger adults, but their choice of a larger age range (18–44-year-old) may have masked any effect. However, an age effect was not found in a subsequent study with a narrower age range (15–24) [16]. Similarly, no age effect was found in a Dutch adult cohort [14]. In contrast, the Baltimore cohort, which focused on incident suicidal ideation and attempt, reported higher transition rates in younger adults (18–29) [15]. Younger age is associated with higher impulsivity, peaking around 16 years-old (with some interindividual heterogeneity) [23] and impulsivity is associated with a higher risk of suicide attempt in young people [24]. Additional individual and societal factors may contribute to higher acting out in young suicidal people including contagion and media effects [25]. A potential higher transition rate in younger individuals needs more investigation.

We furthermore report that lower socioeconomic status (including specific professional categories such as manual workers, employees, intermediate professions, and low income) was independently associated with higher suicidal transition rates. This association has previously been reported [3, 15, 26]. The causal mechanisms are likely multifactorial, including, but not limited to, higher stress, more developmental difficulties and mental issues, lower access to care and cascade effects [27]. Reversing socioeconomic disadvantages may help to prevent suicidal acts.

These findings may have scientific, clinical and policy implications. First, our findings support the distinction between suicidal ideation and act, and the avoidance of the generic term “suicidality”. Therefore, research should continue to explore the social, clinical, psychological, and biological mechanisms of the suicidal transition and distinguish them from those facilitating the emergence of suicidal ideation. Current models of suicidal behavior, such as the “ideation to action” theories [28, 29], make a distinction between processes leading to ideation (e.g., pain, hopelessness) and those leading to action (e.g., access to lethal means, personal history of suicidal act). Although we did not examine risk factors of suicidal ideation here, our study reported factors more specifically associated to action. Second, the low rate of suicidal transition explains the limited value of suicidal ideation to predict a suicidal act. Identifying the factors and mechanisms of the suicidal transition may improve prediction, follow-up, and organization of care with a patient in crisis. Third, preventative strategies targeting all people with suicidal ideation, e.g. specific hotlines for people in suicidal crisis, may have limited effects on the prevention of suicide attempts and suicide death as most suicidal people will not “transition”. People in suicidal crisis may more effectively benefit from an easy access to primary care, combined with a good psychological and psychiatric evaluation. Studies have shown that up to 60% of people who died from suicide had seen their physician or presented to an emergency room the month before their death [30], suggesting potential improvement possibly through training. In contrast, focusing on people who have attempted suicide as currently promoted by some preventative strategies such as “VigilanS” in France [31], may be relevant. Meta-analyses have shown that the risk of repeated self-harm within one year of self-harm hospitalization is 16.3% while the risk of suicide is 1.6% [32]. Finally, improvement in socioeconomic status may have significant effect on the prevention of suicidal acts. All these suggestions are speculative, and a formal assessment of national strategies (both suicide-related and socioeconomic) is needed.

Finally, no clinical condition was statistically associated with higher suicidal transition rates in multivariable analyses, although a trend toward significance was found for daily smoking. Contrary to expectations, we did not find a higher transition rate in heavy drinkers after controlling for smoking. Similarly, a current depressive episode was not related to higher suicidal transition as previously reported by some [3, 7], but not all studies [14, 15].

In conclusion, we report a mean 7.7% suicidal transition rate, with yearly fluctuations, in a large sample of French adults aged 18 to 75. Nine out of ten suicidal people, therefore, do not attempt suicide in the general population. Variations across time may not be related to temporal trends in transition. Higher suicidal transition was independently associated with a history of previous suicide attempt, younger age and lower socioeconomic status. Clinicians examining suicidal patients should be aware of these findings when organizing care and follow-up. Extra vigilance for those with a personal history of suicide attempt is highly recommended.

## Data Availability

Data from Santé Publique France’s Health Barometer is available through individual data sharing agreements.
